# Avian Species Richness in Relation to Intensive Forest Management Practices in Early Seral Tree Plantations

**DOI:** 10.1371/journal.pone.0043290

**Published:** 2012-08-15

**Authors:** Jay E. Jones, Andrew J. Kroll, Jack Giovanini, Steven D. Duke, Tana M. Ellis, Matthew G. Betts

**Affiliations:** 1 Statistics, Mathematics, and Operations Research, Weyerhaeuser NR, Federal Way, Washington, United States of America; 2 Timberlands Technology, Weyerhaeuser NR, Federal Way, Washington, United States of America; 3 Department of Forest Ecosystems and Society, Oregon State University, Corvallis, Oregon, United States of America; Lakehead University, Canada

## Abstract

**Background:**

Managers of landscapes dedicated to forest commodity production require information about how practices influence biological diversity. Individual species and communities may be threatened if management practices truncate or simplify forest age classes that are essential for reproduction and survival. For instance, the degradation and loss of complex diverse forest in young age classes have been associated with declines in forest-associated Neotropical migrant bird populations in the Pacific Northwest, USA. These declines may be exacerbated by intensive forest management practices that reduce hardwood and broadleaf shrub cover in order to promote growth of economically valuable tree species in plantations.

**Methodology and Principal Findings:**

We used a Bayesian hierarchical model to evaluate relationships between avian species richness and vegetation variables that reflect stand management intensity (primarily via herbicide application) on 212 tree plantations in the Coast Range, Oregon, USA. Specifically, we estimated the influence of broadleaf hardwood vegetation cover, which is reduced through herbicide applications, on bird species richness and individual species occupancy. Our model accounted for imperfect detection. We used average predictive comparisons to quantify the degree of association between vegetation variables and species richness. Both conifer and hardwood cover were positively associated with total species richness, suggesting that these components of forest stand composition may be important predictors of alpha diversity. Estimates of species richness were 35–80% lower when imperfect detection was ignored (depending on covariate values), a result that has critical implications for previous efforts that have examined relationships between forest composition and species richness.

**Conclusion and Significance:**

Our results revealed that individual and community responses were positively associated with both conifer and hardwood cover. In our system, patterns of bird community assembly appear to be associated with stand management strategies that retain or increase hardwood vegetation while simultaneously regenerating the conifer cover in commercial tree plantations.

## Introduction

Landscapes dedicated to timber commodity production are often managed for multiple objectives, including retention of populations and communities of native organisms, maintenance of ecosystem services, and the sustainable flow of commodities [1], [2]. However, relatively few studies quantify the relationship between specific practices that are employed over large areas and species and community responses, despite the fact that even modest changes to current practices may provide substantial ecological benefits [3], [4], [5].

Rising global demand for wood and pulp products has led to intensification of forest management practices and a commensurate increase in concern about how these practices influence biological diversity [6], [7]. Intensive forest management practices typically include clearcutting, rapid regeneration of single-species conifer stands, and chemical control of competing vegetation, resulting in truncated successional stages [8]. In particular, herbicide applications are designed to suppress naturally regenerating vegetation, including hardwood and deciduous broadleaf plants that are important components of biological diversity [9], [10]. As a result, species that rely on early seral conditions may be vulnerable, as their preferred habitat is reduced in quality and available for only short periods of time [11], [12].

The potential link between stand management practices and population declines of broadleaf-associated avian species has been observed around the globe where forest management has favored planted conifers over naturally regenerated broadleaf species [13], [14], [15]. For example, forest practices on private lands in the Pacific Northwest (PNW), USA, coupled with reduced harvest rates and the promotion of late seral forest on federal lands, have resulted in a decline in the amount of high quality, early seral habitat available for Neotropical migrant bird species [16], [17], [18]. Breeding Bird Survey results from 1966 to 2007 in the South Pacific Rainforest Bird Conservation Region indicate declining trends for several Neotropical migrant bird species that breed in early seral forest [19], [20]. Species more strongly associated with broadleaf forest are declining at the greatest rates [18].

**Figure 1:**
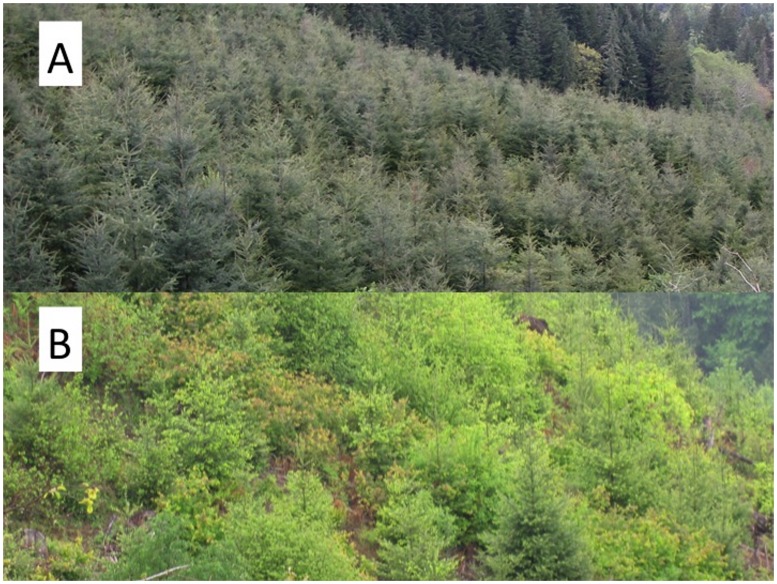
The range of vegetation cover resulting from herbicide control of competing vegetation in conifer plantations. A 10-year old Douglas-fir plantation with <5% hardwood cover (A) and a 7-year old Douglas-fir plantation with ∼50% hardwood cover (B). Our results indicate that species richness was positively associated with both hardwood and conifer cover.

We evaluated relationships between stand management intensity and avian species responses in the Oregon Coast Range, USA. Specifically, we examined how differences in species-level occupancy and community richness varied across gradients in four important measures of vegetation composition: coniferous, broadleaf, deciduous broadleaf, and hardwood vegetation (see Table S1 for dominant species). Herbicide control is directed at broadleaf, deciduous broadleaf, and hardwood vegetation (Figure 1). By examining avian association with these three stand characteristics, we quantify, albeit indirectly, evidence for the biological impact of herbicide control. We expected that leaf-gleaning species would respond more strongly to reductions in hardwood and deciduous broadleaf vegetation, given their reliance on these vegetation features for foraging and breeding [12], [21]. As a result, we summarized leaf-gleaner responses both separately and as part of the overall avian community.

## Results

**Figure 2:**
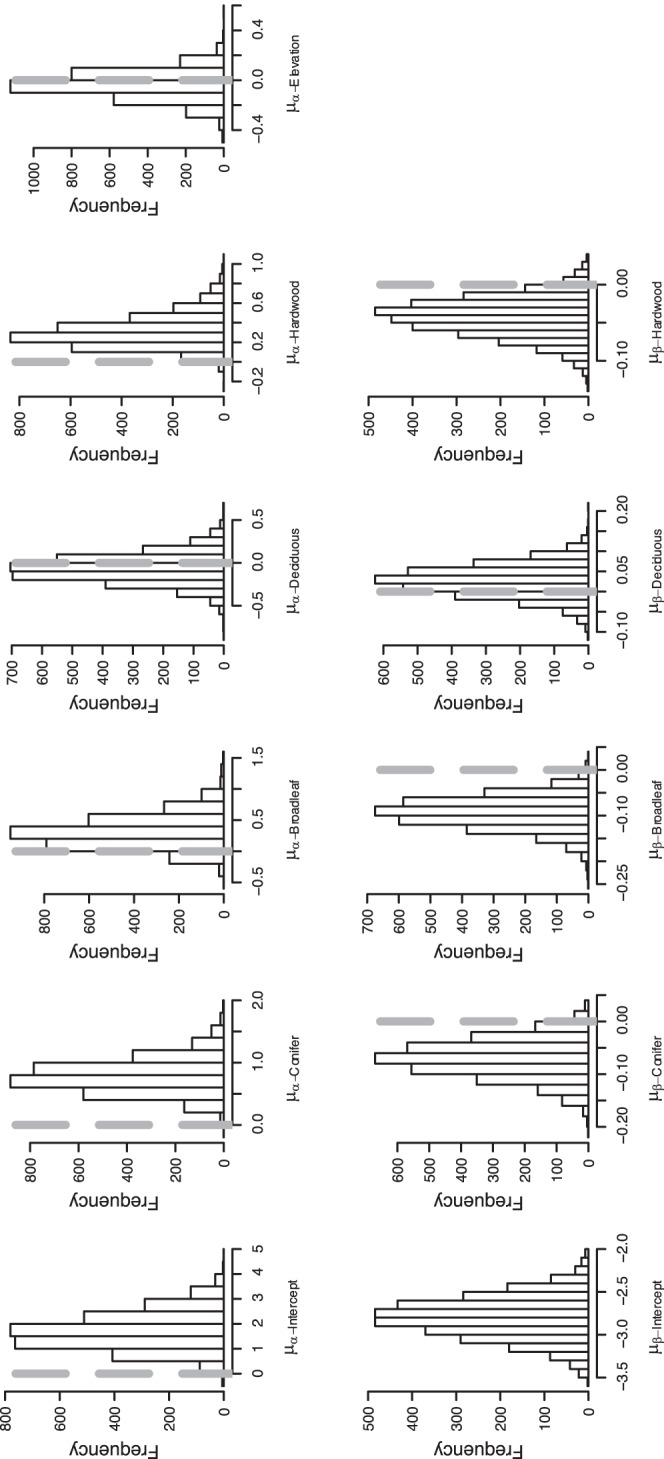
Posterior distributions of the population level hyper-parameter means for occupancy (top row) and detection (bottom row) covariates, Oregon Coast Range, USA, 2008–2009. Dashed gray line indicates zero. Covariates were centered and scaled for analysis.

Population-level mean occupancy was positively associated with cover of both coniferous and hardwood vegetation (Figure 2). This result indicates that on average, occupancy probabilities across all species in this study tended to be higher in stands with greater percentages of conifer and hardwood cover. In contrast, posterior intervals for the community hyper-parameters for elevation and deciduous broadleaf cover were centered near zero, indicating little overall population-level effect of these covariates.

**Figure 3:**
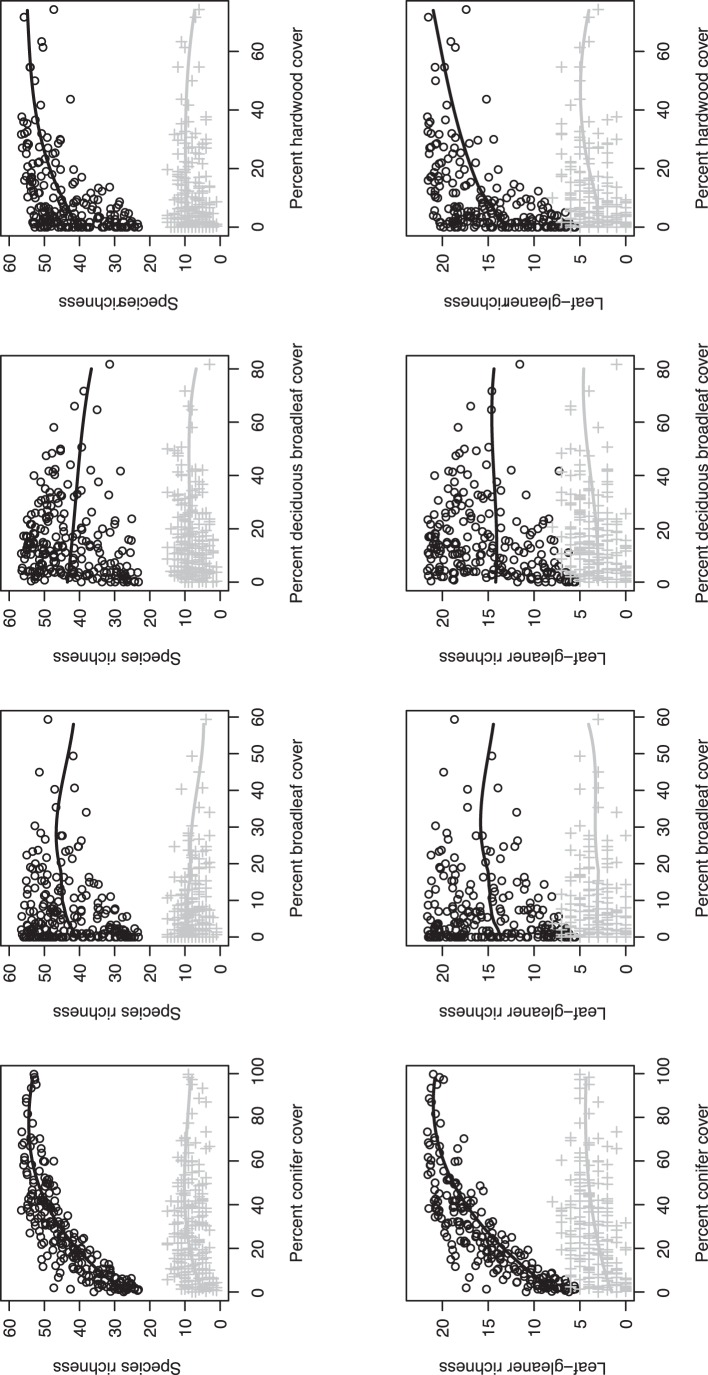
Total estimated species richness (top row) and leaf-gleaner richness (bottom row), based on elevation (not shown) and conifer, broadleaf, deciduous broadleaf, and hardwood vegetation cover, on 212 early seral forest stands, Oregon Coast Range, USA, 2008–2009. Estimates are from a model that incorporates variation in species detection (circles) and a model that ignores variation in species detection (crosses). Solid lines show smoothed trends in expected richness while holding all other covariates at their median values.

We estimated species richness in each of the 212 stands using the community model (circles, Figure 3). Congruent with previous results (12), the response to hardwood cover was stronger than for broadleaf or deciduous broadleaf cover, suggesting a greater biological value of this vegetation feature to breeding birds. Importantly, estimates of total species and leaf-gleaner richness were 35–80% lower if we ignored variation associated with detection probability (crosses, Figure 3).

**Figure 4:**
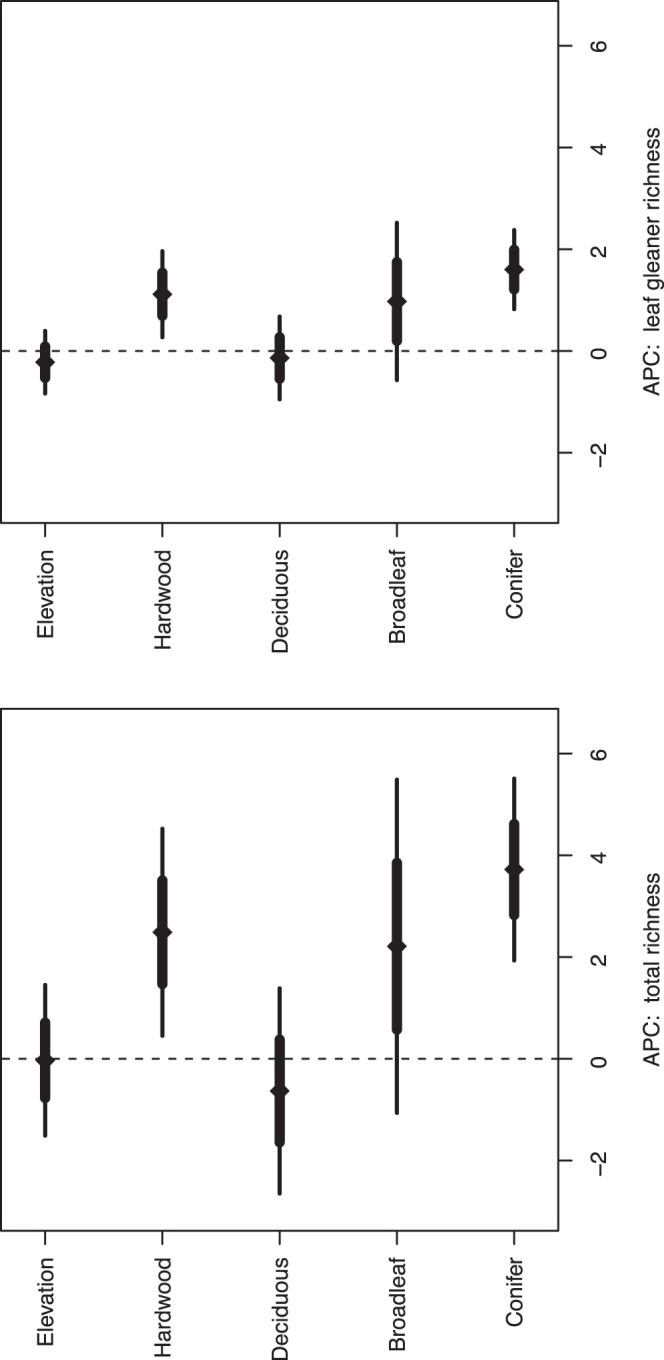
Average predictive comparisons (+/−1 and 2 standard errors) for the association between total species richness and leaf-gleaner richness and elevation (m), percent cover of hardwoods, deciduous, deciduous broadleaf, and conifer vegetation, Oregon Coast Range, USA, 2008–2009. Comparisons were calculated based on a modification of methodology described in Gelman and Pardoe [66].

Conifer cover showed the largest contribution to species richness in our model, with an average predictive comparison (APC) of approximately 3.7 (SE = 0.9) (Figure 4). This result indicates that for two sites with otherwise similar levels of other covariates, we expect ∼3.7 additional species will occur on the site with 10% greater conifer cover (hereafter, all predictive comparisons for vegetation variables correspond to a 10% difference in cover). The APC for hardwood and broadleaf cover were 2.5 and 2.2, respectively, although the estimate for broadleaf cover indicated substantial uncertainty in this estimate. The APC for deciduous broadleaf cover suggested a negative association of avian richness with this covariate although the estimate was small and one SE included 0.

The difference in estimate magnitude for each cover type suggests that vegetation composition, rather than total cover, may drive species richness. For example, the difference in richness associated with a 10% difference in conifer cover is substantially larger than that associated with a similar difference in deciduous cover. If total cover were the primary driver of richness, we would expect all of the cover APC values (i.e., broadleaf, deciduous broadleaf, hardwood, conifer) to be similar, and to be the same as the APC for total cover – i.e., the association between richness and total cover would be independent of how cover was obtained. The APC for a 10% difference in total cover (not shown in Figure 4), as calculated from our model, was 2.3 additional species (SE = 0.4), a value that is less than our estimate for conifer cover, but greater than for deciduous cover.

Leaf gleaners showed similar, but reduced, trends for each of the model inputs (Figure 4) with APCs of 1.6, 1.1 and 1.0 for conifer, hardwood, and broadleaf cover, respectively. For leaf gleaner total richness, the APC for total cover was 1.0 (SE = 0.2).

Estimates of individual species’ occupancy probabilities across all study sites (at median covariate levels) ranged from 0.23–0.99. For many species, detection probabilities were low, ranging from 0.01–0.65. We include posterior summaries of occupancy and detection, as well as estimates and 95% credible intervals (the Bayesian equivalent of a confidence interval) [22] for occupancy and detection covariates, in Table S2.

Both conifer and hardwood cover were more strongly associated with species occupancy than broadleaf or deciduous broadleaf cover (Table S2). For example, the occupancy of 56 of 64 species increased positively with conifer cover (i.e., >200% increase in odds of occurrence across the sampled values of conifer cover), 4 species showed a decrease in occupancy probability (>200% decrease in odds of occurrence across the sampled values of conifer cover), and 4 species showed no change (less than 200% change in odds of occurrence in either direction). Among species with >10 detections, white-crowned sparrow *Zonotrichia leucophrys* and willow flycatcher *Empidonax traillii* showed the strongest negative and positive responses, respectively, to conifer cover.

The occupancy of fifty-six of 64 species showed a strong positive response to increases in hardwood cover (i.e., >200% increase in odds of occurrence across the sampled values of hardwood cover), 1 species showed a decrease in occupancy probability (>200% decrease in odds of occurrence across the sampled values of conifer cover), and 7 species showed no change (less than 200% change in odds of occurrence in either direction). Among species with >10 detections, White-crowned sparrow *Zonotrichia leucophrys* and black-throated gray warbler *Setophaga nigrescens* showed the strongest negative and positive responses, respectively, to hardwood cover.

**Figure 5:**
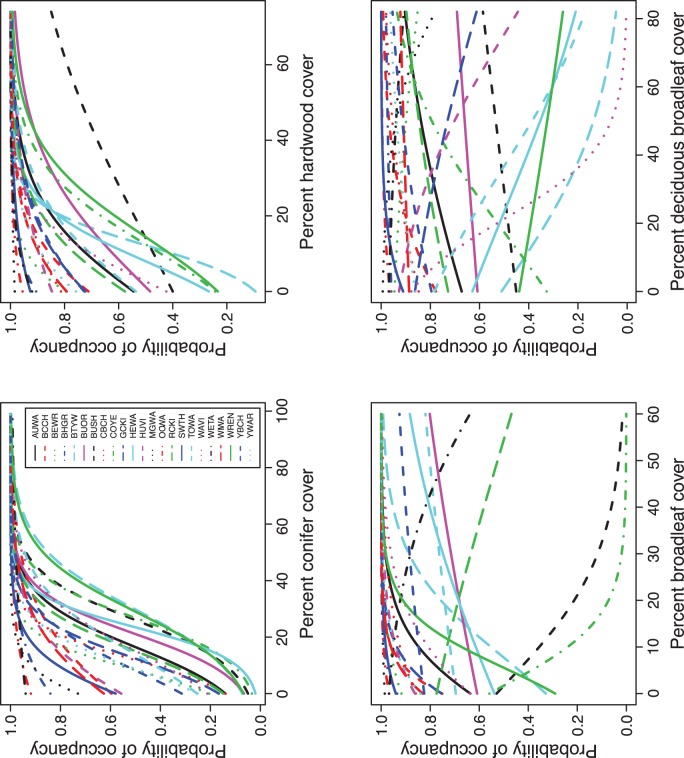
Mean occupancy probabilities of 23 species of leaf-gleaning birds based on percent cover of conifers, broadleaf, deciduous broadleaf, and hardwood vegetation, Oregon Coast Range, USA, 2008–2009. Species codes are in Table S2. Estimates in each panel were calculated while holding all other covariates constant at their median values.

Mean posterior occupancy probability for each of the 23 leaf-gleaning species was positively associated with both conifer and hardwood cover (Figure 5 and Table S2). However, some species that showed positive associations with hardwood cover were negatively associated with deciduous broadleaf cover. For example, bushtit *Psaltriparus minimus*, common yellowthroat *Geothlypis trichas*, ruby-crowned kinglet *Regulus calendula*, and western tanager *Piranga ludoviciana* occupancy probability estimates were negatively associated with broadleaf cover. Similarly, Bewick’s wren *Thryomanes bewickii*, black-throated gray warbler *Setophaga nigrescens*, golden-crowned kinglet *Regulus satrapa*, hermit warbler *Setophaga occidentalis*, Hutton’s vireo *Vireo huttoni*, MacGillivray’s warbler *Geothlypis tolmiei*, Townsend’s warbler *Setophaga townsendi*, warbling vireo *Vireo gilvus*, western tanager, and wrentit *Chamaea fasciata* were negatively associated with deciduous broadleaf cover (Figure 4 and Table S2). Although the number of detections of these species varied substantially (2–197), our use of the community model allowed us to estimate the responses of the individual species to the vegetation covariates, which would have been challenging with more conventional single-species models.

## Discussion

Stand regeneration practices that limit plant community composition and structural complexity may reduce habitat quality and amount for early seral forest species [23]. However, our results indicate that efforts may be undertaken in intensive forest management to mitigate impacts of plantation forestry. Bird species richness was strongly and positively related to the total amount of hardwood cover. Though these results are correlative, they suggest that maintaining non-coniferous vegetation within a stand should have useful conservation benefits. These results concur with similar work from the same study region that examined abundance of birds caught in mistnets (12) and productivity of individual species [24]. Interestingly, species richness also showed a substantial positive association with conifer cover that was larger than the estimated association with total cover. Given that high rates of seedling survival are often positively associated with conifer cover in plantations in our region [25], these results suggest that successful stand regeneration is not necessarily incompatible with conservation goals across the stand ages we observed.

We expect that food availability and predation avoidance were the primary reasons for the large effects of both hardwood and conifer cover on bird species richness. That is, stands with higher vegetation cover will likely support larger invertebrate numbers per unit area than stands with reduced vegetation cover and, consequently, support enhanced reproductive success and survival [26], [27], [28]. Also, increased amounts of vegetation cover may provide high quality nesting sites and reduce efficiency of predators that target nests and adults [29], [30].

Given that we evaluated occupancy as the primary response, we note two important caveats from our study. First, although we did not find strong or consistent effects of deciduous broadleaf and broadleaf cover on avian species richness, the abundance of individual bird species may have been reduced if they relied on specific plant species that were in turn reduced by regeneration practices [10]. Second, our sampling method counted singing males and we do not know how regeneration practices may have influenced measures of demographic performance, including nesting success, productivity, or individual survival. Given the intensification of forest management both regionally and globally [6], [31], evaluation of demographic responses across a gradient of regeneration intensity could provide managers with powerful tools to integrate conservation of biological diversity with commodity production [32], [33].

Our inferences are confined to the stand ages (0–18 years old) evaluated in our study, and we cannot address changes in avian species richness beyond that time frame. Indeed, both theory and empirical evidence indicate that plantations with closed conifer canopies will have simplified vertical structure and plant diversity, leading to reductions in biological diversity [8]. As a result, the positive relationship between diversity and conifer canopy cover that we observed in our study may not be maintained as plantations age. Importantly, the correlation between hardwood and conifer cover across our study sites is not consistent over time. In the initial years of forest succession, these variables are positively correlated. However, after eight years, they become negatively correlated (See Methods and Figure S1). Thus, in the initial years of plantation development, the two key factors influencing diversity coincide. Later, they become decoupled, likely due to competition between conifers and hardwoods as the canopy closes [34], [35]. This indicates a potential trade-off in the later years of stand development between the relative contributions to richness of conifers versus hardwoods.

Our study was a natural experiment rather than a manipulative experiment, thus, our ability to sample the full gradient in hardwood cover was constrained by the characteristics of existing early seral stands within our study area. Stands with high hardwood cover are rare (current state policy requires reduction of these competing hardwood species), so we were not able to predict avian diversity in stands with >60% hardwood cover.

Previous studies have found strong correlations between vegetation structure (i.e., vertical distribution of foliage) and avian species richness [36], but less support exists for a relationship with vegetation composition [37], [38], [39], [40], [41], [42]. Our results raise the questions of whether vegetation composition did not exert an independent effect on richness in these studies, or whether variation associated with the detection process masked an effect? For example, if we had not modeled the detection process, our estimates of species richness would have been much lower (Figure 3) and substantially less correlated with the variables we examined. Vegetation structure is known to interfere with the detection process during avian sampling [43], [44], in which case making unbiased inferences about how vegetation structure influences ecological responses (e.g., occupancy or abundance) will be challenging if the detection process is ignored. In general, we found very low detection probabilities for many species (Table S2), a result that is consistent with findings from recent studies of forest bird communities [45], [46]. Although a diverse suite of statistical tools now exist to incorporate variation from the detection process into ecological modeling [47], [48], we encourage investigators to consider this issue in the design stage of research projects [49].

We did not evaluate the effect of plant species richness on avian species richness, as we assumed that total hardwood/broadleaf cover was associated with hardwood and broadleaf species richness in our study area [12]. The response of higher-level taxa to plant species richness has a long-standing theoretical basis [50], as well as recent empirical support [51], although this response could be a function of both greater resource availability and more complex vegetation structure [52]. For instance, although we did not predict the strong association between conifer cover and avian species richness, conifer species richness clearly did not account for this relationship as study stands were dominated by a single conifer species.

We could not determine if species richness on our study sites was in response to vegetation composition per se (i.e., growth of hardwood and conifer cover) or to community assembly following disturbance (i.e., forest harvesting) [53]. Tree regeneration in our study area is generally very rapid, and older plantations are likely to have high conifer cover. We think that among stand variation in trajectories of plant succession is the most likely explanation for our observations, although we cannot preclude the possibility that intrinsic biological processes influenced the responses that we observed. For instance, bird richness might be higher in older stands because of the time required for species to re-colonize following disturbance (i.e., timber harvest) rather than conifer growth per se. However, given the high vagility of most bird species in our system, it seems unlikely that dispersal limitation is the primary driver of our results.

Land use intensification plays a critical role in provisioning rapidly growing human populations and has potentially severe consequences for the conservation of native biological diversity [54], [55]. Species richness is frequently measured in research studies and management programs to assess community responses to anthropogenic disturbances [56], [57], but reasons exist for considering species richness as only a preliminary, and potentially not very informative, assessment. First, species occupancy (e.g., at the stand level) may remain unchanged even if demographic measures such as survival and reproduction are changing, a critical result for management of individual populations. Second, and more importantly, species richness can remain constant despite substantial changes in community membership. For example, Harvey and Villalobos [58] reported bird assemblages that were equally abundant, speciose, and diverse in agro-forestry systems compared to unmanaged forests. However, the species composition of these assemblages was highly modified, with fewer forest-dependent species, more open area species, and different dominant species. In these cases, the critical question is not what species are present, but the roles they play in ecosystem functioning [55].

## Methods

### Study Area & Bird Observations

We collected our data over a 2-year period (May–July in 2008 and 2009) in 212 forested stands located in the western hemlock zone in western Oregon, USA. We selected stands using a stratified sampling design that represented available gradients in stand age 0–8 years) and proportion of hardwood tree cover (estimated visually upon initial encounter; 0–75%). We did not use a stratified-random design because stands with >10% hardwood cover were relatively rare in our study area. We sampled all stands with >10% cover that we could locate on state or private land within Benton and Polk Counties, Oregon, USA. We received written or verbal permission to sample sites from all private and public landowners involved in the study. No formal permits were required. This approach allowed us to sample across a broad gradient of hardwood cover (0–60%) in existing Douglas-fir plantations, which we considered to be a proxy for intensive forest management. Sites were not broadcast-burned prior to planting of conifer seedlings and treatment with herbicides.

The avian community was sampled using a single fixed-radius point count station in each stand [59]. Point count stations were located >50 m from clearly identifiable forest edges (e.g., roads, forest of different age classes). The average distance between points was 685 m (SE = 41). We conducted two, 5-minute counts on separate occasions, spaced >10 days apart, between 0530 and 1000 hours. Counts were not conducted in the rain or when wind exceeded 20 km/h. We recorded all male birds seen or heard within a 50-m radius as present. At each point count station, we estimated the total cover of coniferous, broadleaf, deciduous broadleaf, and hardwood vegetation (Table S1) in three 3 m-radius circles distributed throughout the 50-m count area (the point center, and 50 m from the point in two random directions). In order to evaluate major broadleaf types separately, we defined deciduous broadleaf cover exclusive of hardwood cover (i.e., total deciduous broadleaf cover minus total hardwood cover) and broadleaf cover exclusive of deciduous broadleaf cover.

Due to the strong correlation between stand age and conifer cover in our study (Pearson correlation coefficient = 0.71; 90% confidence interval = 0.62, 0.80), we were unable to separate effects of stand regeneration (i.e., increasing vegetation cover) and time since disturbance (i.e., forest harvesting) [53]. In general, older stands with low conifer cover are not a deliberate management objective and are rare in our study region. As a result, we present the association of conifer cover and species richness rather than stand age per se. In addition, we evaluated the association between conifer and hardwood cover across the stand ages that we sampled (Figure S1). We found that the correlation between stand conifer and hardwood cover differed by stand age, with a positive correlation for stands less than 8 years of age (0.27; 90% confidence interval = 0.12, 0.41), and a negative correlation for stands greater than 8 years of age (−0.20; 90% confidence interval = −0.38,−0.02).

### Analysis

We used the Dorazio-Royle community occupancy model [48] to examine the relationships between stand management intensity and avian species responses in the Oregon Coast Range, USA. We used the model to estimate species level covariate effects, as well as population level measures of occupancy, including species richness [45], [48]. Following Russell et al. [60], we do not account for the contribution of unobserved species in our population estimates, instead conditioning on the set of observed breeding species in our study.

We let 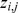 denote true occupancy status, in which 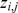 = 1 if species *i* occupies site *j* for the study interval, or 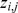 = 0 otherwise. The occupancy state is taken to be a Bernoulli random variable, 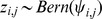, where 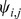 is the probability that species *i* occupies site *j*. We take species detection to again follow a Bernoulli distribution: 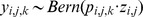, where 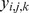 is 1 if the species *i* is detected at site *j* during visit *k*, or 0 otherwise. Note that under this parameterization, the probability of detecting the species *i* at site *j* will be zero if the species does not occupy site *j*, since 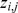 = 0.

We modeled species-specific occupancy probabilities as a function of the 4 vegetation covariates, plus site-level elevation, using a logit link function. The effect of elevation was not a focus of our study, but prior studies have found associations between bird species richness and elevation and we wanted to control for this source of variation [61], [62]:
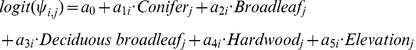
(1)


We modeled species-specific detection probabilities as a function of the 4 vegetation covariates only:
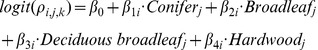
(2)


Each covariate was centered and scaled prior to analysis. We included each of our primary variables of interest in both the occupancy and detection models. This approach is necessary to separate occupancy effects, which are of primary interest, from potential detection effects; otherwise, our estimates of richness could be biased.

Under the hierarchical community model, we assume that the species-specific effects for a given parameter are drawn from a common normal distribution, e.g., that
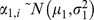
for parameter 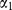
of species *i,* where the mean and variance are population-level hyper-parameters. This population-level distribution provides a summary of community response, both in terms of the mean behavior as well as the variability in behavior. The extent to which information is shared across species depends on both the degree of uniformity across the population, as estimated by the population-level parameters, and the amount of information available for each species. For species for which we are less certain of the parameter estimates, e.g., those with low detection probabilities, estimates will tend to shrink toward the population mean value.

All computations were performed using WinBUGS [63] called from R [64] using package R2WinBUGS [65]. We ran 3 chains of length 100,000 each, with a burn-in of 50,000 and 1/50 thinning. We assessed convergence using the Gelman-Rubin statistic [22] and visual inspection of the chains, with both measures indicating a reasonable assumption of convergence. We provide WinBUGS code for this model in Text S1.

Species richness is not modeled directly in the Dorazio-Royle community occupancy model and we are not aware of any existing methods for quantifying the association between model covariates and species richness within their framework. Past approaches have focused on visual displays of estimated richness [45], [56]. Such displays are useful, but inference is indirect and not quantitative. Here, we adopt the use of average predictive comparisons [66] to quantify directly the association (and uncertainty) between *predicted* species richness and each model covariate. Predictive comparisons evaluate the difference in expected response for a unit difference in an input covariate, using the fitted model, and averaging over the distribution of all other covariates. We extend this approach to species richness by summing over the species-specific predictions to obtain averaged expected differences in species count. For our dataset 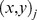, 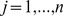, we denote our input of interest *u*, and all other inputs *v*, such that *x = (u,v)*, where n is the number of sites. We let 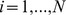, be the index of species, where N is the total number of observed species. We estimated the average predictive comparison for species richness using Equation 3

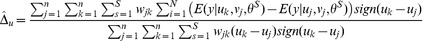
(3)


A set of 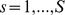 simulations were sampled from the posterior distribution. We calculated predictive comparisons for all model inputs, treating each in turn as the input of interest. Standard errors for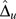 are estimated as described in Gelman and Pardoe [66], and account for the uncertainty in model parameter estimates, while treating all covariates as fixed. We note that unlike the richness estimator described in Dorazio and Royle [48], the approach described here is based on the model prediction, and does not directly consider the observed occupancy status of each site.

The predictive comparison approach may also be extended to post-hoc combinations of inputs, in addition to the individual model inputs. For a linear function *f* of *q* inputs of interest, we can estimate the average predictive comparison for species richness as in equation (4):
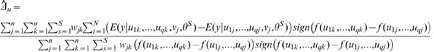
(4)


We used Equation (4) to examine the association between total cover of all 4 vegetation classes and both total species richness and leaf-gleaner richness. We provide all code used to estimate average predictive comparisons in Text S1.

## Supporting Information

Figure S1 The correlation between conifer cover and hardwood cover by stand age class (split into two groups by the median stand of 8 years), for 212 forest stands, Oregon Coast Range, USA, 2008–2009.
(EPS)

Table S1 Common plant species with classification as conifer (*), broadleaf, deciduous broadleaf, or hardwood vegetation, Oregon Coast Range, USA, 2008–2009.
(DOC)

Table S2 Median species-specific estimates and 95% credibility intervals for occupancy and detection and parameter estimates and 95% credibility intervals for elevation and vegetation covariates on intensively managed forest stands, Oregon Coast Range, USA, 2008–2009. We included the total number of observations for each species and if species were classified as leaf-gleaners.
(XLS)

Text S1 WinBUGS code for hierarchical community model and average predictive comparisons of species richness.
(DOC)
