# Supplementary figures and images for: Avian Species Richness in Relation to Intensive Forest Management Practices in Early Seral Tree Plantations
Source: PLoS One. 2012 Aug 15;7(8):e43290. doi: 10.1371/journal.pone.0043290 (PMC3419709; doi:10.1371/journal.pone.0043290)

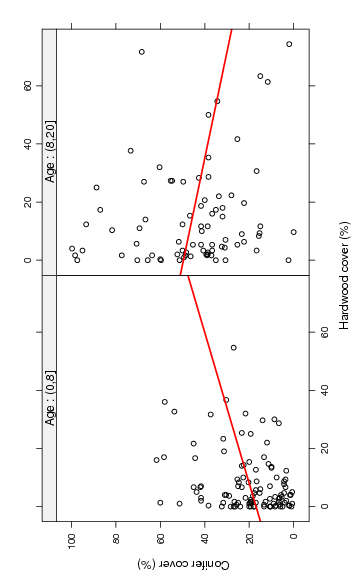

Supplement: Figure S1 — The correlation between conifer cover and hardwood cover by stand age class (split into two groups by the median stand of 8 years), for 212 forest stands, Oregon Coast Range, USA, 2008–2009. (EPS) [file pone.0043290.s001.tif]
