# Supplementary material for: Avian Species Richness in Relation to Intensive Forest Management Practices in Early Seral Tree Plantations
Source: PLoS One. 2012 Aug 15;7(8):e43290. doi: 10.1371/journal.pone.0043290 (PMC3419709; doi:10.1371/journal.pone.0043290)
Supplement: Table S1 — Common plant species with classification as conifer (*), broadleaf, deciduous broadleaf, or hardwood vegetation, Oregon Coast Range, USA, 2008–2009. (DOC) [file pone.0043290.s002.doc]

**Appendix S1: Common plant species within early seral stands, Oregon Coast Range, USA, 2008-2009, with classification as conifer (*), broadleaf, deciduous broadleaf, or hardwood**.

| **Common name** | **Latin name** | **Broadleaf** | **Deciduous broadleaf** | **Hardwood** |
| --- | --- | --- | --- | --- |
| Douglas-fir* | *Pseudotsuga menziesii* |  |  |  |
| True fir* | *Abies spp.* |  |  |  |
| Hemlock* | *Tsuga heterophylla* |  |  |  |
| Pine* | *Pinus spp.* |  |  |  |
| Red alder | *Alnus rubra* |  | X | X |
| Bigleaf maple | *Acer macrophyllum* |  | X | X |
| Vine maple | *Acer circinatum* |  | X | X |
| Elderberry | *Sambucus spp.* |  | X | X |
| Cherry | *Prunus spp.* |  | X | X |
| Dogwood | *Cornus nuttallii* |  | X | X |
| Cascara | *Rhamnus purshiana* |  | X | X |
| Hazel | *Corylus cornuta* |  | X | X |
| Serviceberry | *Amelanchier alnifolia* |  | X | X |
| Indian plum | *Oemleria cerasiformis* |  | X | X |
| Oak | *Quercus spp.* |  | X | X |
| Chinkapin | *Chrysolepis chrysophylla* | X |  | X |
| Madrone | *Arbutus menziesii* | X |  | X |
| Poison oak | *Toxicodendron diversilobum* |  | X |  |
| Rose | *Rosa spp.* |  | X |  |
| Black-/Raspberry | *Rubus spp.* |  | X |  |
| Huckleberry | *Vaccinium spp.* |  | X |  |
| Snowberry | *Symphoricarpus albus* |  | X |  |
| Currant | *Ribes spp.* |  | X |  |
| Oceanspray | *Holodiscus discolor* |  | X |  |
| Scotch broom | *Cytisus scoparius* | X |  |  |
| Salal | *Gaultheria shallon* | X |  |  |
| Oregon grape | *Mahonia nervosa* | X |  |  |
| Manzanita | *Arctostaphylos spp.* | X |  |  |
| Ceanothus | *Ceanothus velutinus* | X |  |  |
| Rhododendron | *Rhododendron macrophyllum* | X |  |  |
