# Supplementary material for: Avian Species Richness in Relation to Intensive Forest Management Practices in Early Seral Tree Plantations
Source: PLoS One. 2012 Aug 15;7(8):e43290. doi: 10.1371/journal.pone.0043290 (PMC3419709; doi:10.1371/journal.pone.0043290)
Supplement: Text S1 — WinBUGS code for hierarchical community model and average predictive comparisons of species richness. (DOC) [file pone.0043290.s004.doc]

**Appendix S4:** WinBUGS code for hierarchical community model and R code for calculating Average Predictive Comparisons, Oregon Coast Range, USA, 2008-2009.

**WinBUGS code for hierarchical community model**

model

{

mu.a0 ~ dnorm(0.00000E+00, 0.333)

mu.a1 ~ dnorm(0.00000E+00, 0.5)

mu.a2 ~ dnorm(0.00000E+00, 0.5)

mu.a3 ~ dnorm(0.00000E+00, 0.5)

mu.a4 ~ dnorm(0.00000E+00, 0.5)

mu.a5 ~ dnorm(0.00000E+00, 0.5)

mu.b0 ~ dnorm(0.00000E+00, 0.333)

mu.b1 ~ dnorm(0.00000E+00, 0.5)

mu.b2 ~ dnorm(0.00000E+00, 0.5)

mu.b3 ~ dnorm(0.00000E+00, 0.5)

mu.b4 ~ dnorm(0.00000E+00, 0.5)

sig2.a0 ~ dgamma(2, 0.5)

sig2.a1 ~ dgamma(3, 0.5)

sig2.a2 ~ dgamma(3, 0.5)

sig2.a3 ~ dgamma(3, 0.5)

sig2.a4 ~ dgamma(3, 0.5)

sig2.a5 ~ dgamma(3, 0.5)

sig2.b0 ~ dgamma(2, 0.5)

sig2.b1 ~ dgamma(3, 0.5)

sig2.b2 ~ dgamma(3, 0.5)

sig2.b3 ~ dgamma(3, 0.5)

sig2.b4 ~ dgamma(3, 0.5)

tau.a0 <- 1/sig2.a0

tau.a1 <- 1/sig2.a1

tau.a2 <- 1/sig2.a2

tau.a3 <- 1/sig2.a3

tau.a4 <- 1/sig2.a4

tau.a5 <- 1/sig2.a5

tau.b0 <- 1/sig2.b0

tau.b1 <- 1/sig2.b1

tau.b2 <- 1/sig2.b2

tau.b3 <- 1/sig2.b3

tau.b4 <- 1/sig2.b4

for (i in 1:n) {

a0[i] ~ dnorm(mu.a0, tau.a0)

a1[i] ~ dnorm(mu.a1, tau.a1)

a2[i] ~ dnorm(mu.a2, tau.a2)

a3[i] ~ dnorm(mu.a3, tau.a3)

a4[i] ~ dnorm(mu.a4, tau.a4)

a5[i] ~ dnorm(mu.a5, tau.a5)

b0[i] ~ dnorm(mu.b0, tau.b0)

b1[i] ~ dnorm(mu.b1, tau.b1)

b2[i] ~ dnorm(mu.b2, tau.b2)

b3[i] ~ dnorm(mu.b3, tau.b3)

b4[i] ~ dnorm(mu.b4, tau.b4)

for (j in 1:J) {

logit(psi[j, i]) <- a0[i] + a1[i] * ELEVM[j] + a2[i] *

CONIFER[j] + a3[i] * BROADsansDEC[j] + a4[i] *

DECsansHWD[j] + a5[i] * HWD[j]

mu.psi[j, i] <- min(0.99999, max(1.00000E-05, psi[j,

i]))

Z[j, i] ~ dbern(mu.psi[j, i])

Zleaf[j, i] <- Z[j, i] * leafgleaners[i]

for (k in 1:K) {

logit(p[j, k, i]) <- b0[i] + b1[i] * CONIFER[j] +

b2[i] * BROADsansDEC[j] + b3[i] * DECsansHWD[j] +

b4[i] * HWD[j]

mu.p[j, k, i] <- min(0.99999, max(p[j, k, i],

1.00000E-05)) * Z[j, i]

X[j, k, i] ~ dbern(mu.p[j, k, i])

}

}

}

for (j in 1:J) {

Nsite[j] <- sum(Z[j, ])

Nleaf[j] <- sum(Zleaf[j, ])

}

}

**R code for calculating Average Predictive Comparisons using a ‘bugs’ object.**

**# APC for each individual model covariate**

apcFun <- function(parlist, covdata, ndraws, sig.v){

n <- nrow(covdata)

nsim <- length(parlist[[1]])

s.index <- sample(1:nsim, ndraws, replace=TRUE)

out <- matrix(nrow=ndraws, ncol=5)

for(s in 1:ndraws){

print(s)

D.conifer.num <- 0

D.conifer.den <- 0

D.broad.num <- 0

D.broad.den <- 0

D.dec.num <- 0

D.dec.den <- 0

D.hwd.num <- 0

D.hwd.den <- 0

D.elev.num <- 0

D.elev.den <- 0

theta.s <- cbind(parlist$a0[s.index[s],], parlist$a2[s.index[s],], parlist$a3[s.index[s],],

parlist$a4[s.index[s],], parlist$a5[s.index[s],], parlist$a1[s.index[s],])

for(i in 1:n){

#print(i)

covi <- covdata[i,]

for(j in 1:n){

# CONIFER

covj <- covi

covj$CONIFER <- covdata$CONIFER[j]

w.ij <- 1/(1+t(as.numeric(covdata[j,-1] - covdata[i,-1])) %*% sig.v[2:5,2:5] %*% (as.numeric(covdata[j,-1] - covdata[i,-1])))

sign.ij <- sign(covdata$CONIFER[j] - covdata$CONIFER[i])

D.conifer.num <- D.conifer.num + (w.ij * (sum(1/(1+exp(-theta.s %*% c(1, as.numeric(covj))))) - sum(1/(1+exp(-theta.s %*% c(1, as.numeric(covi)))))) * sign.ij)

D.conifer.den <- D.conifer.den + (w.ij * sign.ij * (covdata$CONIFER[j] - covdata$CONIFER[i]))

# BROAD

covj <- covi

covj$BROADsansDEC <- covdata$BROADsansDEC[j]

w.ij <- 1/(1+t(as.numeric(covdata[j,-2] - covdata[i,-2])) %*% sig.v[c(1,3:5),c(1,3:5)] %*% (as.numeric(covdata[j,-2] - covdata[i,-2])))

sign.ij <- sign(covdata$BROADsansDEC[j] - covdata$BROADsansDEC[i])

D.broad.num <- D.broad.num + (w.ij * (sum(1/(1+exp(-theta.s %*% c(1, as.numeric(covj))))) - sum(1/(1+exp(-theta.s %*% c(1, as.numeric(covi)))))) * sign.ij)

D.broad.den <- D.broad.den + (w.ij * sign.ij * (covdata$BROADsansDEC[j] - covdata$BROADsansDEC[i]))

# DEC

covj <- covi

covj$DECsansHWD <- covdata$DECsansHWD[j]

w.ij <- 1/(1+t(as.numeric(covdata[j,-3] - covdata[i,-3])) %*% sig.v[c(1:2,4:5),c(1:2,4:5)] %*% (as.numeric(covdata[j,-3] - covdata[i,-3])))

sign.ij <- sign(covdata$DECsansHWD[j] - covdata$DECsansHWD[i])

D.dec.num <- D.dec.num + (w.ij * (sum(1/(1+exp(-theta.s %*% c(1, as.numeric(covj))))) - sum(1/(1+exp(-theta.s %*% c(1, as.numeric(covi)))))) * sign.ij)

D.dec.den <- D.dec.den + (w.ij * sign.ij * (covdata$DECsansHWD[j] - covdata$DECsansHWD[i]))

# HWD

covj <- covi

covj$HWD <- covdata$HWD[j]

w.ij <- 1/(1+t(as.numeric(covdata[j,-1] - covdata[i,-4])) %*% sig.v[c(1:3,5),c(1:3,5)] %*% (as.numeric(covdata[j,-4] - covdata[i,-4])))

sign.ij <- sign(covdata$HWD[j] - covdata$HWD[i])

D.hwd.num <- D.hwd.num + (w.ij * (sum(1/(1+exp(-theta.s %*% c(1, as.numeric(covj))))) - sum(1/(1+exp(-theta.s %*% c(1, as.numeric(covi)))))) * sign.ij)

D.hwd.den <- D.hwd.den + (w.ij * sign.ij * (covdata$HWD[j] - covdata$HWD[i]))

# ELEVM

covj <- covi

covj$ELEVM <- covdata$ELEVM[j]

w.ij <- 1/(1+t(as.numeric(covdata[j,-5] - covdata[i,-5])) %*% sig.v[1:4,1:4] %*% (as.numeric(covdata[j,-5] - covdata[i,-5])))

sign.ij <- sign(covdata$ELEVM[j] - covdata$ELEVM[i])

D.elev.num <- D.elev.num + (w.ij * (sum(1/(1+exp(-theta.s %*% c(1, as.numeric(covj))))) - sum(1/(1+exp(-theta.s %*% c(1, as.numeric(covi)))))) * sign.ij)

D.elev.den <- D.elev.den + (w.ij * sign.ij * (covdata$ELEVM[j] - covdata$ELEVM[i]))

}

}

out[s,1] <- D.conifer.num / D.conifer.den

out[s,2] <- D.broad.num / D.broad.den

out[s,3] <- D.dec.num / D.dec.den

out[s,4] <- D.hwd.num / D.hwd.den

out[s,5] <- D.elev.num / D.elev.den

print(out[s,])

}

out

}

# Note that ‘covdat’ is the name of the original (scaled) dataset

covdat.apc <- covdat[,c("CONIFER", "BROADsansDEC", "DECsansHWD", "HWD", "ELEVM")]

sig.v <- cov(covdat.apc)

system.time(apc <- myFun(parlist=fit$sims.list, covdata=covdat.apc, ndraws=200, sig.v=sig.v) )

# NOTE 1: the first argument is the ‘sims.list’ from a fitted ‘bugs’ object in R, using package

# ‘R2WinBUGS’. i.e., we assume the WinBUGS model shown above was fit in R using

# the ‘bugs’ function.

# NOTE 2: this took about 18 hours to run on a 2.26GHz Core i7 Processor with 2GB of RAM.

# NOTE 3: APC estimates were rescaled after calculation

# NOTE 4: The same function was used for leaf-gleaner APC; the ‘parlist’ argument used only

# leaf-gleaner species parameters.

**# APC for Total Cover**

myFun2 <- function(parlist, covdata, ndraws, sig.v){

n <- nrow(covdata)

nsim <- length(parlist[[1]])

s.index <- sample(1:nsim, ndraws, replace=TRUE)

#out <- matrix(nrow=ndraws, ncol=5)

out <- numeric(ndraws)

for(s in 1:ndraws){

print(s)

D.total.num <- 0

D.total.den <- 0

theta.s <- cbind(parlist$a0[s.index[s],], parlist$a2[s.index[s],], parlist$a3[s.index[s],],

parlist$a4[s.index[s],], parlist$a5[s.index[s],], parlist$a1[s.index[s],])

for(i in 1:n){

#print(i)

covi <- covdata[i,]

for(j in 1:n){

# Total

covj <- covi

covj[,1:4] <- covdata[j,1:4]

w.ij <- 1/(1+t(as.numeric(covdata[j,5] - covdata[i, 5])) %*% sig.v[5,5] %*% (as.numeric(covdata[j,5] - covdata[i,5])))

sign.ij <- sign(covdata$Total[j] - covdata$Total[i])

#print(as.numeric(covj))

#print(theta.s)

D.total.num <- D.total.num + (w.ij * (sum(1/(1+exp(-theta.s %*% c(1, as.numeric(covj[-6]))))) - sum(1/(1+exp(-theta.s %*% c(1, as.numeric(covi[-6])))))) * sign.ij)

D.total.den <- D.total.den + (w.ij * sign.ij * (covdata$Total[j] - covdata$Total[i]))

}

}

out[s] <- D.total.num / D.total.den

print(out[s])

}

out

}

covdat.apc$Total <- with(covdat.apc, c((CONIFER*10+25 ) + (BROADsansDEC*5+3.3) + (DECsansHWD*9 + 12) + (HWD*5+10))/10) # Covariates must be rescaled to calculate ‘Total’

system.time(apc.total <- myFun2(parlist=fit$sims.list, covdata=covdat.apc, ndraws=200, sig.v=sig.v) )

# Summarize results for 10%-point difference in cover or 100m difference in elevation

apply(apc, 2, mean) * c(10/10, 10/5, 10/9, 10/5, 100/70) # APC mean for each model covariate

apply(apc,2,sd) * c(10/10, 10/5, 10/9, 10/5, 100/70) # APC std. error for each model covariate

mean(apc.total) # APC mean for total cover

sd(apc.total) # APC std. error for total cover
